# Diffusion-induced lithium isotopic heterogeneity in olivines from peridotites of an oceanized mantle lithosphere at the Yunzhug ophiolite (central Tibet)

**DOI:** 10.1038/s41598-024-54616-6

**Published:** 2024-02-19

**Authors:** Ren-Deng Shi, Guang-hao Cui, Qi-Shuai Huang, Xiao-Xiao Huang, Haibo Zou, Xiaohan Gong, Jing-Sui Yang

**Affiliations:** 1 State Key Laboratory of Tibetan Plateau Earth System, Environment and Resources (TPESER), Institute of Tibetan Plateau Research, Chinese Academy of Sciences, Beijing, 100101 China; 2 University of Chinese Academy of Sciences, Beijing, 100049 China; 3 College of Geosciences and Engineering, North China University of Water Resources and Electric Power, Zhengzhou, 450045 China; 4 Department of Geosciences, Auburn University, Auburn, AL 36849 USA; 5 School of Ocean Sciences, China University of Geosciences, Beijing, 100083 China; 6 School of Earth Sciences and Engineering, Nanjing University, Nanjing, 210023 China

**Keywords:** Li isotopes, Heterogeneity, Seawater diffusion, SCLM peridotite, Tibetan Yunzhug ophiolite, Geochemistry, Petrology

## Abstract

This paper reports lithium concentrations and isotopic compositions of olivines in the oceanized subcontinental lithospheric mantle (SCLM) peridotites of the Tibetan Yunzhug ophiolite. The results show systematic Li isotope changes with distance from the rim of olivine grains. δ^7^Li values of olivine in dunites decrease from + 10.46 to + 1.33‰ with increasing distance to olivine rim from 26.15 to 124.71 μm. A negative correlation of δ^7^Li and Li content in olivine from dunite and harzburgite indicates recent diffusive ingress of Li into the peridotites. The extremely heavy Li isotopic composition requires the seawater or seawater alteration endmember in the mixing model, and reveals Li diffusion from seawater into olivine. As in dunites, olivines in a harzburgite sample show similar variations in δ^7^Li as a function of distance from the grain rim (e.g., 6.01 to 1.73 in sample 14YZ13). We suggest that the behavior of Li in the oceanized SCLM peridotites may be controlled by Li diffusion from seawater, as Li activity in the liquid state is higher than the solid state in transporting Li through the olivines in the peridotites. This study supports that seawater Li diffusion is one of the important factors for the heterogeneity of mantle Li isotopes in ophiolites.

## Introduction

High-temperature processes are generally considered as important factors of mantle isotope heterogeneity, such as the interaction of lithosphere and mantle plume and the recycling of oceanic lithosphere at different stages of Wilson cycles^1–7^. High-temperature processes are often used to explain the isotopic heterogeneity of mantle peridotite in ophiolites. Such interpretaions of isotopic heterogeneity in ophiolites are certainly suitable for lithophile and siderophile element tracers such as Sm–Nd, Lu–Hf, Re–Os and Mg–Fe high-temperature isotopic systems^8^, but are not necessarily suitable for fluid-mobile Li in ophiolites. Lithium isotopes are useful not only for tracing crust-mantle recycling in subduction system, but also for studying low temperature fluid-rock interactions, due to the high solubility of lithium in melts/fluids, the large relative mass difference between ^7^Li and ^6^Li, and the higher diffusivity of ^6^Li relative to ^7^Li.^9,10^. Li isotopes can be strongly fractionated at low temperatures with δ^7^Li values of + 31‰ in seawater^11^. Thus, the surficial process also could change Li isotope composition of oceanic lithospheric rocks on the seafloor by seawater-rock exchange at low temperature. For example, seawater-basalt exchange could produce Li enrichment and a δ^7^Li increase in altered basalts^12,13^, and during serpentinization of oceanic peridotites, ‘relict’ olivine and pyroxenes generally have lower Li concentrations and higher δ^7^Li values as a result of Li diffusion in seawater^14^. Thus, the near-surface geological process is an important factor in producing Li isotope heterogeneity in the mantle sequence of ophiolite.

Ophiolites are generally considered as pieces of suboceanic lithospheric mantle that have been thrust onto the edges of continental plates^15,16^. Mantle peridotite is a diagnostic unit of ophiolites. The peridotite from the Yunzhug ophiolite (Tibet) is a product of oceanization of subcontinental lithospheric mantle during the incipient rifting of the Gondwana continent, tectonically exposed on the ocean floor by large-scale detachments^17^. We selected olivines from peridotites of this ophiolite for a detailed in-situ Li isotope study to understand the ophiolitic mantle heterogeneity at the early stage of the Wilson cycle.

## Geological setting and sample descriptions

**Figure 1:**
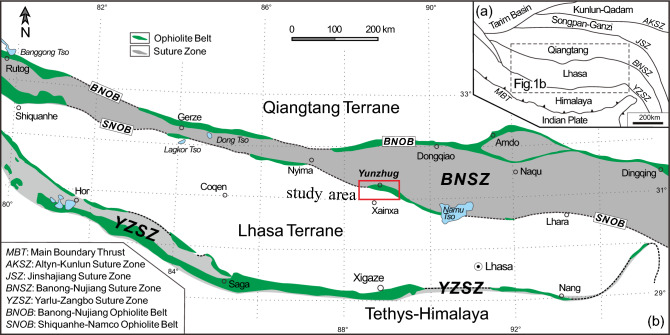
(**a**) Tectonic framework of Tibet and adjacent India, showing position of Bangong-Nujiang Suture Zone (BNSZ); (**b**) tectonic map of BNSZ showing the relationship between BNSZ and Bangong-Nujiang Ophiolite Belt (BNOB) and Shiquanhe-Namco Ophiolite Belt (SNOB) and other ophiolite belt (e.g. Naqu), and location of the Yunzhug ophiolite in Xainxa area^16^.

The Yunzhug ophiolite, located northeast of Xainxa county in central Tibet, is in the middle part of the Shiquanhe-Namo ophiolite belt which is the southern boundary of the Bangong-Nujiang suture zone (BNSZ). The BNSZ extends approximately 1200 km across central Tibetan Plateau and separates the Lhasa terrane to the south and the Qiangtang terrane to the north. The Yunzhug ophiolite is mainly composed of ophiolitic blocks and thick sequences of Jurassic flysch and associated volcanic rocks. The ophiolitic massifs are up to 200 km long in the middle sector of BNSZ referred to as the Northern Tibetan Neo-Tethyan ophiolites^18–21^ (Fig. 1).

Mantle peridotite as the main component of the Yunzhug ophiolite crops out about 200 km^2^, accounting for more than 90% in volume of the ophiolite. Associated mafic lavas are minor. The isolated ophiolitic massifs oriented in a NWW-SEE direction, are surrounded by Paleozoic–Mesozoic strata, and contain many autoclastic and exotic blocks. Small amounts of radiolarian cherts associated with the lavas are between Late Triassic and Late Cretaceous in age. The predominant serpentinized harzburgites are crosscut by centimeter- to decimeter-scale pyroxenite and meter-scale banded and laminated dunite. Compared with the classical Penrose ophiolite sequence in the relative positions of the various lithologies, the Yunzhug ophiolite differs from the traditional Cyprus ophiolite^22^ and Oman ophiolite^23^. However, it is comparable with the Alpine-Apennine ophiolites^24^, and displays strong similarities with the lithospheric structure of the Iberia-Newfoundland margin characteristic of young passive continental margins^17^.

The mantle peridotites studied here are mainly harzburgites with minor dunites that were slightly serpentinized. Fresh minerals are preserved widely in most harzburgites and locally in some dunites. The fresh mineral grains are mainly olivines with Fo values ranging from 91.8 to 93.8. Orthopyroxene (Opx) is generally found as 3 to 5 mm porphyroclasts. Compositionally, the Opx is magnesian (Mg# = 91.7–93.3). Their Al_2_O_3_ (0.45–1.55 wt%) and CaO content (usually 0.5–1.0 wt%, and less than 1.40 wt%) are consistent with those Opx from on-craton garnet lherzolites. Clinopyroxene (Cpx) has lower Al_2_O_3_ and Na_2_O, and elevated Cr_2_O_3_ contents with an embryonic oceanic-crust affinity. The low Os isotopic compositions (^187^Os/^188^Os = 0.11301–0.12374; T_RD_ = 2.30–0.54 Ga) and high Mg# values of whole rocks and high Fo in olivine indicate that the peridotites originated from ancient subcontinental lithospheric mantle (SCLM). The fine-grained mineral clusters reflect modification of the peridotite massif, representing the oceanization process during the incipient rifting of the Gondwana continent^17^.

## Lithium concentrations and isotopic compositions in olivines

**Figure 2:**
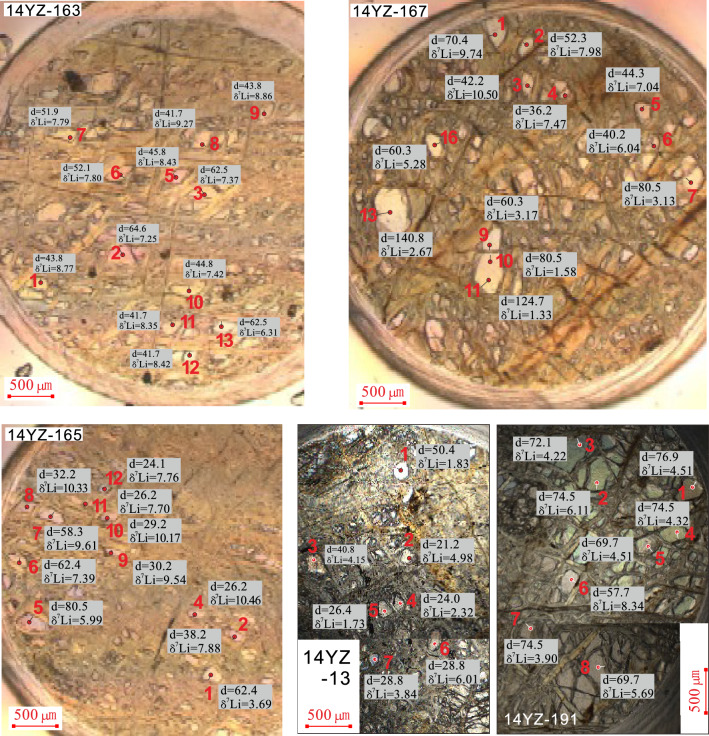
Scanned image of mounts showing olivine grains in the Yunzhug ophiolitic harzburgite and dunite for in situ Li isotope analysis, d = the distance from the rim of each grain.

**Figure 3:**
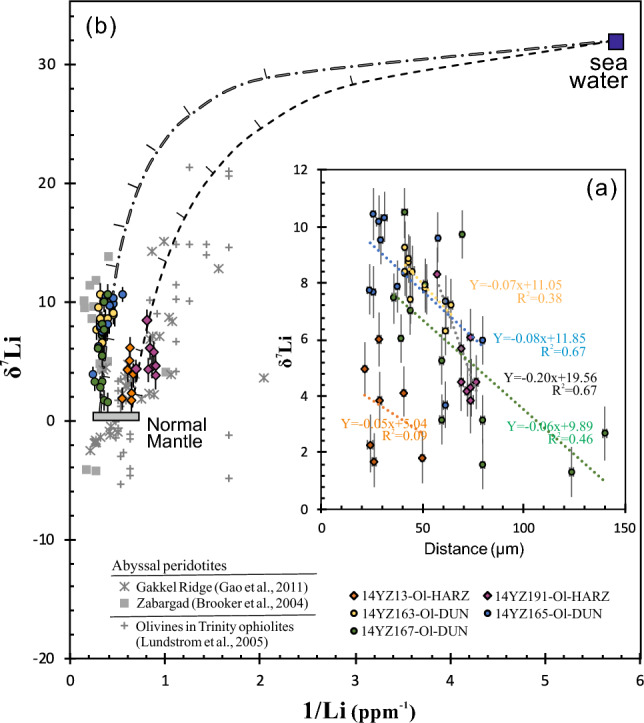
(**a**) Plot of δ^7^Li values versus the distance from the rim of olivine grain; (**b**) δ^7^Li values and Li content of olivine plot along the mixing line between seawater and subcontinental lithospheric mantle (close to the normal mantle).

Lithium concentrations and isotopic compositions (δ^7^Li) for both dunites and harzburgites are reported in Table 1 and plotted in Figs. 2 and 3a as a function of a distance from the rim of each olivine grain. The detailed information of δ^7^Li and the distance from the rim (d) are shown in Fig. 3a. Lithium contents of the olivine grains analyzed here are different between dunite and harzburgites. Comparable to the estimate of the upper mantle Li content (2.00 ppm)^25,26^, olivines in dunites have generally higher Li concentrations ranging from 2.13 to 3.55 ppm (with the exception of one grain at 1.80 ppm) than those in harzburgite (1.11–1.83 ppm). There are large variations in Li isotopic compositions of olivines. The correlation between Li isotopic composition and Li content of olivine in dunite is better than that in harzburgite. δ^7^Li values of olivine plot along the mixing line between seawater and subcontinental lithospheric mantle (Fig. 3b).

**Table 1:** Li content, δ^7^Li values of olivines from the Yunzhug ophiolitic harzburgite and dunite, central Tibet.

Sample @ spot	Rock type	δ7Li	1SE	Li (ppm)	1SE	Distance from rim (μm)
14YZ-13OL@1	Harzburgite	1.83	0.90	1.83	0.010	50.4
14YZ-13OL@2		4.98	0.90	1.62	0.009	21.6
14YZ-13OL@3		4.15	0.89	1.63	0.008	40.8
14YZ-13OL@4		2.32	1.00	1.54	0.008	24.0
14YZ-13OL@5		1.73	0.98	1.57	0.008	26.4
14YZ-13OL@6		6.01	0.96	1.58	0.007	28.8
14YZ-13OL@7		3.84	0.98	1.49	0.007	28.8
14YZ-191OL@1	Harzburgite	4.51	0.96	1.17	0.005	76.9
14YZ-191OL@2		6.11	0.97	1.19	0.006	74.5
14YZ-191OL@3		4.22	0.97	1.45	0.007	72.1
14YZ-191OL@4		4.32	1.02	1.23	0.006	74.5
14YZ-191OL@5		4.51	1.02	1.12	0.006	69.7
14YZ-191OL@6		8.34	0.96	1.27	0.006	57.6
14YZ-191OL@7		3.90	1.20	1.11	0.005	74.5
14YZ-191OL@8		5.69	0.98	1.15	0.006	69.7
14YZ-163OL@1	Dunite	8.77	0.92	2.17	0.017	43.8
14YZ-163OL@2		7.25	0.91	2.73	0.019	64.6
14YZ-163OL@3		7.37	0.84	2.93	0.013	62.5
14YZ-163OL@5		8.43	0.91	2.77	0.018	45.8
14YZ-163OL@6		7.80	0.90	2.76	0.016	52.1
14YZ-163OL@7		7.79	0.85	2.54	0.024	51.9
14YZ-163OL@8		9.27	0.90	3.20	0.020	41.7
14YZ-163OL@9		8.86	0.86	2.15	0.012	43.8
14YZ-163OL@10		7.42	0.85	3.32	0.022	44.8
14YZ-163OL@11		8.35	0.85	3.10	0.020	41.7
14YZ-163OL@12		8.42	0.87	2.43	0.017	41.7
14YZ-163OL@13		6.31	0.84	2.90	0.018	62.5
14YZ-165OL@1	Dunite	3.69	0.83	3.81	0.025	62.4
14YZ-165OL@2		7.88	0.91	2.41	0.018	38.2
14YZ-165OL@4		10.46	0.91	1.80	0.010	26.1
14YZ-165OL@5		5.99	0.85	3.24	0.016	80.5
14YZ-165OL@6		7.39	0.92	2.79	0.013	62.4
14YZ-165OL@7		9.61	0.89	2.10	0.011	58.3
14YZ-165OL@8		10.34	0.88	2.60	0.017	32.2
14YZ-165OL@9		9.54	0.88	2.37	0.015	30.2
14YZ-165OL@10		10.17	0.95	2.13	0.015	29.2
14YZ-165OL@11		7.70	0.91	3.02	0.017	26.1
14YZ-165OL@12		7.76	0.89	3.06	0.021	24.1
14YZ-167OL@1	Dunite	9.74	0.88	2.72	0.016	70.4
14YZ-167OL@2		7.98	0.88	2.81	0.017	52.3
14YZ-167OL@3		10.50	0.89	2.50	0.015	42.2
14YZ-167OL@4		7.47	0.89	2.65	0.015	36.2
14YZ-167OL@5		7.04	0.86	2.91	0.017	44.3
14YZ-167OL@6		6.04	0.86	3.31	0.019	40.2
14YZ-167OL@7		3.13	0.86	3.55	0.022	80.5
14YZ-167OL@9		3.17	0.88	2.86	0.017	60.3
14YZ-167OL@10		1.58	0.88	2.74	0.017	80.5
14YZ-167OL@11		1.33	0.89	2.49	0.015	124.7
14YZ-167OL@13		2.67	0.94	3.02	0.018	140.8
14YZ-167OL@16		5.28	0.89	2.84	0.017	60.3

Interestingly, the Li isotopic composition (δ^7^Li) is negatively correlated with the distance from the grain rim. δ^7^Li values of olivines increase from 1.33 to 10.46‰ with a decreasing distance to the rim from 124.71 to 26.15 μm in dunites (Figs. 2, 3a). This negative correlation is supported by the data in individual olivine grains. In sample 14YZ-167, points 9, 10, 11 represent different distances in an individual olivine grain, and their Li isotopic compositions are negatively correlated with the distance to the rim. Point 9 has the highest δ^7^Li value (3.17) with the shortest distance (60.3 μm), while point 11 has the lowest δ^7^Li value (1.33) with the longest distance (124.7 μm), and the point 10 is intermediate in δ^7^Li value and distance. The correlation between Li isotopic composition and the distance from the grain rim for harzburgites is not as good as that of dunites. Nevertheless, the rim of olivine grains from harzburgites still has higher δ^7^Li value relative to the center of the grains.

## Discussion

Ophiolitic mantle peridotites generally undergo at least three processes. The first stage is partial melting of convective upper mantle or primitive upper mantle to produce early harzburgites or dunites; the second stage is refertilization of the early harzburgites or dunites to form Cpx-free harzburgite and Cpx-bearing lherzolite via melt-rock interaction during seafloor spreading or subduction; and the third stage is serpentinization that has pervasively developed in the ophiolitic mantle sequence^5,26–30^. The Yunzhug ophiolitic mantle peridotites are products of oceanization from ancient subcontinental lithospheric mantle (SCLM)^17^. Li abundance and isotopic composition of SCLM is useful for studying the oceanization processes of the ancient SCLM during the early stage of the Wilson cycle.

Homogeneous reservoirs in the mantle with δ^7^Li around + 4‰ have been estimated from the isotopic signatures of OIB and MORB^25^. The studies of peridotite xenoliths hosted in volcanic rocks is another approach to estimate the Li isotopic composition of the upper mantle^31^. Lithium isotopic compositions in some mantle xenoliths and especially the clinopyroxene display a large range^28^. Recent studies show a narrower range of δ^7^Li in peridotite xenoliths from various tectonic settings^32–34^, and most δ^7^Li values are clustered around + 2–4‰ within the range of the normal mantle value (Fig. 3b). Therefore, it is reasonable to use this value as mantle endmember for binary mixing calculation to reveal the characteristics of the samples. The result presented in Fig. 3a demonstrates correlations between Li concentrations and Li isotopic compositions of the olivines from the Yunzhug ophiolitic peridotites, which can be used to infer the possible geological processes and factors that control the Li isotopic composition of the oceanized SCLM.

Lithium diffusion in solid materials (e.g., Ol, Opx and Cpx) is much slower than in melt or fluids. The diffusion of Li into solid materials from an infinite source of Li, such as a surrounding fluid or melt, can be used to explain the Li isotopic disequilibrium of peridotites. Lithium elemental and isotopic disequilibrium of peridotites is produced by the recent melt/fluid-rock reaction or by the last stage serpentinization, as Li isotopes can be strongly fractionated at low temperatures. During the first stage of partial melting and the second stage of refertilizatiion at high temperatures, Li isotopes should approach equilibrium. During the oceanization of ancient SCLM at the early stage of Wilson cycle, serpentinization is the last process of the formation of the ophiolitic mantle peridotites in the continental margin, where seawater circulates and interacts with peridotites. Lithium is a fluid-mobile element and its isotopes can be strongly fractionated at low temperature. Therefore, Li diffusion between seawater and fresh minerals (i.e., Ol) in peridotites is likely to determine the Li elemental and isotope charateristics of the oceanization SCLM. The marked Li isotopic disequilibrium in the olivines from Yunzhug ophiolitic peridotites is likely the product of such diffusion-driven kinetic isotopic fractionation. This scenario is similar to that of Li isotopic characteristics of minerals in the Zabargad peridotites (Fig. 3b) in the Red Sea^35^, which is produced by the active rifting of continent (future ocean-continent-transition (OCT)-type ophiolite), but differs from the olivines in the abyssal peridotites^36^ and the Trinity ophiolitic peridotites^37^ that represent MOR- and SSZ-type ophiolites^38^, respectively.

**Figure 4:**
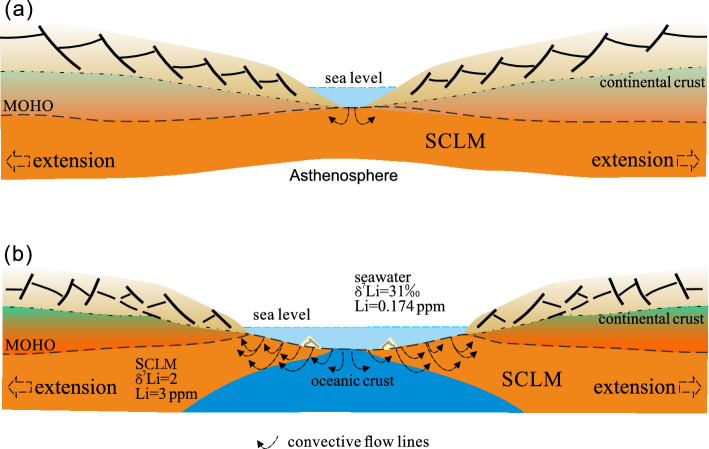
Schematic model showing the processes of seawater Li diffusion into the subcontinental lithospheric mantle (SCLM), (**a**) seawater initiates contact with SCLM at the rifting of continent associated with the upwelling of asthenosphere by detachment at the early stage of the Wilson cycle; (**b**) hydrothermal convection of sea-water through still hot, newly-formed oceanic crust cause further diffusion of seawater Li into the SCLM.

The negative relationship between the δ^7^Li values and the distance from the analysis spot to the rim of the olivines indicates that the Li isotopes in olivines from the Yunzhug oceanization of the ancient SCLM (Fig. 3a) may be controlled by the seawater Li diffusion at the temperature conditions for serpentinization of shallow oceanic crust. As the rifting of ancient continent, the SCLM was gradually exposed to the surface and the hydrothermal convection of seawater permeates through newly formed, still hot oceanic crust and SCLM as shown in Fig. 4, which is supported by previous studies on metamorphism, mineralization and ferromanganoan sediments^39^. The thermal gradient in this single-pass system is greater than 150℃/km^40,41^, and there is enough energy for Li to diffuse from seawater into fresh olivine during the early stage of Wilson cycle. Generally, this diffusion process may involve two steps. The first step occurs prior to serpentinization, where the Li diffusion-induced isotopic heterogeneity took place between the seawater and olivines. The second step occurs after serpentinization, where Li element in the serpentine with seawater Li isotopic features move to relics of olivines. During the first step, ^6^Li and ^7^Li move together into olivine grain by diffusion without obvious isotopic fractionation because of the small isotopic fractionation (the diffusivity ratio D^7^Li/D^6^Li value is close to 1) in the seawater under the low-middle temperatures. The Li ion surrounding the water with hydration shells likely plays an important role in limiting the isotopic heterogeneity associated with diffusion^42^. Therefore, the seawater Li isotopic characteristics can be preserved in the fresh olivine grains by Li ion diffusion.

## Conclusion

The oceanized SCLM peridotites from the Yunzhug ophiolite within the Tibetan Bangong-Nujiang suture zone are characterized by strong Li isotopic disequilibria caused by diffusion of Li from seawater to rocks. Because Li diffuses rapidly at high temperatures during partial melting and melt-rock interactions in the refractory peridotites, Li isotopes should have approached equilibrium before the mantle peridotites were exposed to the seafloor by the detachment during the first stage of Wilson cycle. When the mantle peridotites emerged on the seafloor at lower temperature, Li diffused from seawater to olivine since the Li is a fluid-mobile element and its activity in seawater is significantly higher than that of olivine under low temperature. This study highlights that Li isotopic heterogeneity in olivines of the oceanized SCLM peridotites can be produced by the surficial processes, such as diffusion from seawater.

### In situ* Li concentration and isotopic compositions*

In situ Li elemental and isotopic compositions of olivine were analyzed using a Cameca IMS- 1280HR SIMS at IGGCAS, following established methods^43^. The samples were coated under vacuum with high-purity gold before the SIMS analysis. The primary oxygen ion beam was accelerated at 13 kV, with an intensity of 25 nA. The elliptical spot was approximately 20 × 30 μm in size. Positive secondary ions were measured on an electron multiplier in pulse counting mode, with a mass resolution (M/DM) of 1500 and an energy slit open at 40 eV without any energy offset. Eighty cycles were measured with counting times of 7 and 2 s for ^6^Li and ^7^Li, respectively. The measured δ^7^Li values are given as δ^7^Li ([(^7^Li/6Li) _sample_ /(^7^Li/^6^Li) _L-SVEC_ − 1] × 1000) relative to units of the standard NIST SRM 8545 (L-SVEC) with ^7^Li/^6^Li = 12.0192. The instrumental mass fractionation (IMF) is expressed in δ^7^Li units: Δi = δ^7^Li_SIMS_ − δ^7^Li_MC-ICPMS_. In-house standards used here include two olivines (06JY31: Mg#_90.3; 06JY34: Mg#_91.5), which were detailed described by Su et al. (2015a)^43^. The olivine 06JY34 is used for those samples with higher Mg# in olivines (sample 14YZ-13; 14YZ-163; 14YZ-165; 14YZ-167;14YZ-191). The olivine standards in this study yielded an average δ^7^Li of 4.51 ± 0.78‰ (06JY31_olivine: n = 10; 1se), 3.33 ± 0.72 ‰ (06JY34_olivine: n = 10; 1se), consistent with the recommended values^40^. Lithium concentrations of the samples were calculated based on ^7^Li^+^ count rates (cps/nA) relative to the standard^43^. The detection limit of Li concentration measurements is < 1 ppb and the analytical uncertainties of the Li isotopic compositions are less than 1.5‰ (1se). Previous studies have suggested a substantial matrix effect on the ^7^Li/^6^Li ratio of olivine measured by SIMS^44^, with δ^7^Li increasing by about 1.0‰ for each mole percent decrease in forsterite component^43^. Thus the measured δ^7^Li in olivines from studied samples were further corrected as suggested.

## Data Availability

Data are available through Mendeley Data at https://data.mendeley.com/datasets/zbfk4p559z/1.
